# Extending *Miscanthus* Cultivation with Novel Germplasm at Six Contrasting Sites

**DOI:** 10.3389/fpls.2017.00563

**Published:** 2017-04-19

**Authors:** Olena Kalinina, Christopher Nunn, Ruth Sanderson, Astley F. S. Hastings, Tim van der Weijde, Mensure Özgüven, Ivan Tarakanov, Heinrich Schüle, Luisa M. Trindade, Oene Dolstra, Kai-Uwe Schwarz, Yasir Iqbal, Andreas Kiesel, Michal Mos, Iris Lewandowski, John C. Clifton-Brown

**Affiliations:** ^1^Department of Biobased Products and Energy Crops, Institute of Crop Science, University of HohenheimStuttgart, Germany; ^2^Institute of Biological, Environmental and Rural Sciences, Aberystwyth UniversityAberystwyth, UK; ^3^The Institute of Biological and Environmental Sciences, University of AberdeenAberdeen, UK; ^4^Department of Plant Breeding, Wageningen UniversityWageningen, Netherlands; ^5^Faculty of Agriculture and Natural Sciences, Konya Food and Agriculture UniversityKonya, Turkey; ^6^Department of Plant Physiology, Russian State Agrarian University - Moscow Timiryazev Agricultural AcademyMoscow, Russia; ^7^German Agrarian CentrePotash, Ukraine; ^8^SchwarzBraunschweig, Germany; ^9^Blankney EstatesBlankney, UK

**Keywords:** *Miscanthus*, novel hybrids, multi-location field trials, establishment, productivity, marginal land

## Abstract

Miscanthus is a genus of perennial rhizomatous grasses with C4 photosynthesis which is indigenous in a wide geographic range of Asian climates. The sterile clone, *Miscanthus* × *giganteus (M*. × *giganteus)*, is a naturally occurring interspecific hybrid that has been used commercially in Europe for biomass production for over a decade. Although, *M*. × *giganteus* has many outstanding performance characteristics including high yields and low nutrient offtakes, commercial expansion is limited by cloning rates, slow establishment to a mature yield, frost, and drought resistance. In this paper, we evaluate the performance of 13 novel germplasm types alongside *M*. × *giganteus* and horticultural “Goliath” in trials in six sites (in Germany, Russia, The Netherlands, Turkey, UK, and Ukraine). Mean annual yields across all the sites and genotypes increased from 2.3 ± 0.2 t dry matter ha^−1^ following the first year of growth, to 7.3 ± 0.3, 9.5 ± 0.3, and 10.5 ± 0.2 t dry matter ha^−1^ following the second, third, and fourth years, respectively. The highest average annual yields across locations and four growth seasons were observed for *M*. × *giganteus* (9.9 ± 0.7 t dry matter ha^−1^) and interspecies hybrid OPM-6 (9.4 ± 0.6 t dry matter ha^−1^). The best of the new hybrid genotypes yielded similarly to *M*. × *giganteus* at most of the locations. Significant effects of the year of growth, location, species, genotype, and interplay between these factors have been observed demonstrating strong genotype × environment interactions. The highest yields were recorded in Ukraine. Time needed for the crop establishment varied depending on climate: in colder climates such as Russia the crop has not achieved its peak yield by the fourth year, whereas in the hot climate of Turkey and under irrigation the yields were already high in the first growing season. We have identified several alternatives to *M*. × *giganteus* which have provided stable yields across wide climatic ranges, mostly interspecies hybrids, and also *Miscanthus* genotypes providing high biomass yields at specific geographic locations. Seed-propagated interspecific and intraspecific hybrids, with high stable yields and cheaper reliable scalable establishment remain a key strategic objective for breeders.

## Introduction

There is an increasing demand for sustainably produced biomass in the growing European bioeconomy but its material and energetic use should not compete with food supply (Lewandowski et al., 2016). Therefore, the additionally required biomass should not be grown on good agricultural land but on land that is economically or bio-physically marginal for food production. According to Allen et al. (2014), there are an estimated 1,350,000 hectares (ha) of such land in Europe that is abandoned from or unsuitable for food crop production and could be preferentially exploited for growing biomass crops.

*Miscanthus* is a genus of high-yielding perennial rhizomatous grasses with C4 photosynthesis. It is considered a promising candidate bioeconomy crop due to the combination of high yields, low input demand, good environmental performance, multiple biomass use options, and the potential to grow on land that is considered marginal for food production (Dohleman and Long, 2009; McCalmont et al., 2015; Lewandowski et al., 2016). *Miscanthus* demonstrates a broad genetic variability in the area of its origin, namely East-Asia (Clifton-Brown et al., 2016). However, this theoretical potential cannot yet be exploited fully in Europe. Currently the industrial use of this crop in Europe is limited to one standard clone *Miscanthus* × *giganteus* (*M*. × *giganteus*; Hodkinson and Renvoize, 2001), a sterile interspecific hybrid propagated vegetatively. Cultivation and yields of *M*. × *giganteus* can be limited by low temperatures in the northern European regions (Clifton-Brown and Lewandowski, 2000) and drought in the southern regions (Hastings et al., 2009a,b). Another limitation to the broader distribution of miscanthus are the high production costs for *M*. × *giganteus* (Lewandowski et al., 2016). Vegetative propagation is an expensive way of establishing the plantations (Xue et al., 2015). Introducing new germplasm from the wild collections is needed to extend the geographical range in which *Miscanthus* can be cultivated and overcome some of the current limitations, and some early selections from European breeding programs should create invaluable knowledge of the “Genotype × Environment” interactions.

Germplasm used in European breeding programs belong mainly to the species *M. sacchariflorus* and *M. sinensis*. To date, their interspecific hybrids, such as *M*. × *giganteus*, are generally higher yielding than the pure species (Davey et al., 2017) in temperate zones. A cold tolerance test with five genotypes showed that certain *M. sinensis* types could withstand lower winter temperatures than *M*. × *giganteus* and *M. sacchariflorus* (Clifton-Brown et al., 2000). In general, *M. sinensis* interspecific hybrids have thinner and shorter stems than *M. sacchariflorus* and their hybrids, which combined lead to lower yields in trials with the scientific standard planting density of 20,000 plants ha^−1^ (Iqbal and Lewandowski, 2014). In the UK and Germany, the miscanthus breeding program led by Aberystwyth over the past decade has focussed on producing interspecific *M. sinensis* × *M. sacchariflorus* hybrids with high yield, cold or other stress tolerance and seed production (Clifton-Brown et al., 2016). As high seed production in interspecific hybrids does not occur naturally in Northern Europe, breeders in the Netherlands have focussed on the genetic improvement of intraspecific hybrids of *M. sinensis* types. Scientific field trials have shown the potential for other *M. sinensis* intraspecies hybrids in drought prone areas (Clifton-Brown et al., 2002). During the past decade, the breadth of *Miscanthus* germplasm available in Europe has been expanded through plant collection trips (Clifton-Brown J. C. et al., 2011; Clifton-Brown J. et al., 2011; Hodkinson et al., 2016). There is tremendous diversity available within the *Miscanthus* genus to exploit, particularly within *M. sinensis* which occurs in the widest climatic range of all *Miscanthus* species. *M. sinensis* types are known to senesce earlier than many tall *M. sacchariflorus* types (Robson et al., 2012). *M. sinensis* generally flowers in North European climates (Jensen et al., 2011), while most *M. sacchariflorus* needs warmer climates to flower before winter (Jensen et al., 2013). Although flowering in the production area potentially increases the invasive risk, this can be mitigated by the manipulation of ploidy to produce sterile triploids (Anderson et al., 2006).

In this paper, we report on a multi-location field plot experiment, where we have tested a range of selected diverse germplasm from the different *Miscanthus* species on a wide climatic gradient spanning Atlantic, continental, and Mediterranean climates. All the germplasm entries for this experiment were selected from breeding nurseries in Northern Europe. Four wild “tall *M. sacchariflorus*” types were selected in Aberystwyth from spaced plants trials planted from the accessions collected in 2006/7 from Eastern Asia. Four *M. sinensis* populations were selected: two from Wageningen University and two from open-pollinated “strong” *M. sinensis* parents selected in Northern Germany. Five interspecies hybrids of *M. sinensis* and *M. sacchariflorus* were selected in a spaced plant breeding nursery in Braunschweig, Germany from progeny of different crosses in 2011.

The overarching objective of this study was to create the understanding needed to extend the range for *Miscanthus* production in Eurasia. We were particularly interested in understanding if *Miscanthus* selected in UK, Netherlands, and Germany could both establish, over-winter and produce an economically viable yield with relatively low temperatures and rainfall in Eastern areas. There is a known opportunity for miscanthus cultivation in Eastern European countries such as Ukraine and Russia where both significant amounts of underused land and a strong local market for the biomass for heat exist. Our expectation was that best performers in terms of yield could be identified in each of the six sites due to environmental specificity: both at level of the germplasm groups and at the level of specific genotypes or populations. It was expected that the performance of some of the novel interspecies and intraspecies hybrids would match or exceed *M*. × *giganteus*, thus providing potential growers and end users with new options. We also believed that the knowledge generated by a multi-location trial approach, containing a wide selection of “relevant” germplasm types, would identify environmental specificity for both the parents and progeny of *M. sinensis* and *M. sacchariflorus*. This G × E information can be used to assist breeders to develop better future hybrids. For the purposes of examining G × E interactions we felt it is was necessary to reduce the number of variables by using a high proportion of clonal selections (genotypes) for 11 of the 15 selections rather than individuals from populations derived from “seed.” If any of these clones proved outstanding, then breeding of seed propagated equivalents would be the logical next step. The four seeded entries (of *M. sinensis* type) would be used to explore if phenotypic variation within a population cross was a significant issue for the future expansion of a crop based on seeded *M. sinensis* hybrids.

Our first hypothesis was that, under the wide range of climate and soil conditions between Stuttgart (Germany), Moscow (Russia), Wageningen (The Netherlands), Adana (Turkey), Aberystwyth (UK), and Potash (Ukraine), significant differences would exist in establishment rate and yield performance of the novel germplasm types. The abiotic stress tolerance traits observed would be used to inform further breeding of future seeded hybrids.

Our second hypothesis was that new selections, heretofore only tested in spaced plant nurseries, could perform as well or better than *M*. × *giganteus* in competitive plot trials in sites with more extreme climates and poorer soils than have been tested to date.

## Materials and methods

### Plant material

Germplasm to evaluate was selected by the breeders at Aberystwyth and Wageningen Universities. The fifteen selections included four genotypes of wild *M. sacchariflorus*, five interspecies hybrids of *M. sacchariflorus* × *M. sinensis*, four *M. sinensis* seed-based population hybrids (two of which were paired crosses, and two open-pollinated) and two triploid standard clones: *M*. × *giganteus* (between *M. sinensis* and *M. sacchariflorus*; Greef and Deuter, 1993) and *M. sinensis* “Goliath” (*M. sinensis* × *sinensis*; Table 1). The origins of the germplasm types or their parents, where known, ranged from 23 to 45 N (Supplementary Table 1). The wild *M. sacchariflorus* type collection sites ranged from 31 to 37 N. Growing season rainfall (April–September) at the known locations of germplasm collection range from 500 to 2000 mm p.a. The mean minimum monthly winter temperatures in these areas ranged from −16 to 12°C. The hybrids OPM-6, 7, 8, and 10 and the *M. sinensis* OPM-11, 12 and 15 were provided by Aberystwyth University and the *M. sinensis* genotypes OPM-13 and 14 were provided by Wageningen University. All hybrids and *M. sinensis* were diploid. Some of the wild *M. sacchariflorus* genotypes were tetraploid (see Supplementary Table 1).

**Table 1:** **Germplasm selected for the multi-location trials**.

**Genotype ID**	**Species**	**Accession details**	**Propagation method**
OPM-1	Sac	Wild Sac	*In vitro*
OPM-2	Sac	Wild Sac	*In vitro*
OPM-3	Sac	Wild Sac	*In vitro*
OPM-4	Sac	Wild Sac	*In vitro*
OPM-5	Hybrid	Wild Sin × Wild Sac hybrid	*In vitro*
OPM-6	Hybrid	Wild Sac × Wild Sin	*In vitro*
OPM-7	Hybrid	Wild Sac × Wild Sin	*In vitro*
OPM-8	Hybrid	Wild Sac × Wild Sin	*In vitro*
OPM-9	Hybrid (Gig)	Wild Sac × Wild Sin	*In vitro*
OPM-10	Hybrid	Wild Sac × Wild Sin	*In vitro*
OPM-11	Sin (Goliath)	Wild Sin × open	*In vitro*
OPM-12	Sin	Wild Sin × open	Seeds
OPM-13	Sin	Sin × Sin	Seeds
OPM-14	Sin	Sin × Sin	Seeds
OPM-15	Sac × Sin × open Sin (open-pollinated hybrid with dominating Sin phenotype and high morphological variability)	(Sac × Sin) × open Sin	Seeds

*Sac, M. sacchariflorus; Sin, M. sinensis; Hybrid, M. sinensis × M. sacchariflorus hybrid. Common clone names added where these exist [e.g., Gig = M. × giganteus, Sin (Goliath) = M. sinensis Goliath]*.

*In vitro* propagation was used to produce “plug” plants in modular trays (Quick Pot 96 38 × 38 × 78 mm, HerkuPlast, Kubern, GmbH, Ering/Inn, Germany) from clones OPM 1–11. Seeded entries (OPM-12–15) were sown in similar trays. OPM-13 and OPM-14 were raised in the Netherlands. OPM-12 and OPM-15 were raised in the UK. All were grown in the glasshouse before hardening off, transportation to and transplantation at the six field trial locations. Hereafter, all the germplasm types are referred to as “genotypes.”

### Field trials

**Figure 1:**
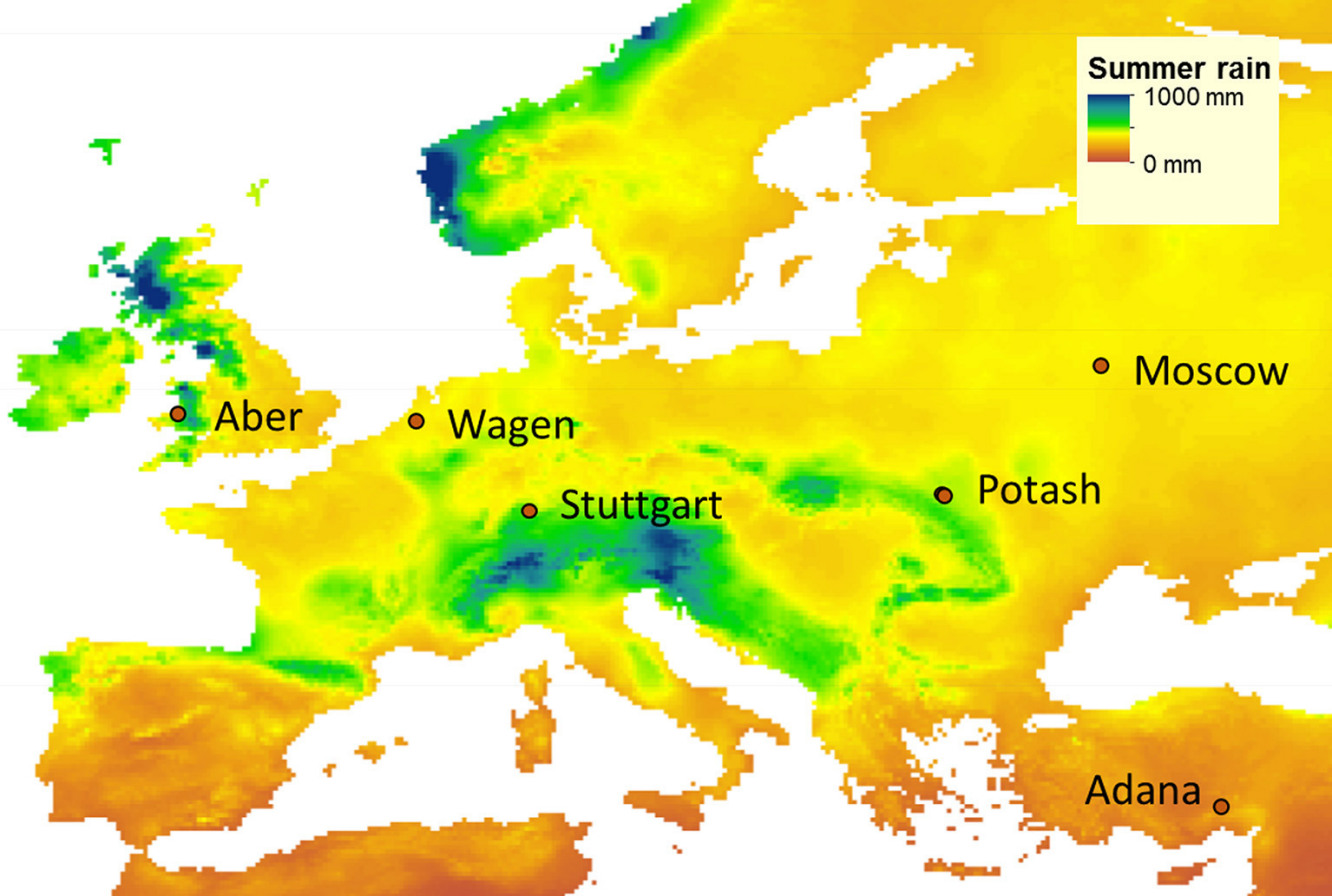
**Location of the field trials established in May 2012: Aberystwyth (Aber; United Kingdom), Wageningen (Wagen; The Netherlands), Stuttgart (Germany), Adana (Turkey), Potash (Ukraine), and Moscow (Russia), and historical summer rainfall map (average of equinox to equinox rainfall from 2010 to 2014 from CRU TS v. 3.24)**.

Between April and May 2012, 15 genotypes (Table 1) were established at six field locations (Figure 1) covering a wide range of environmental conditions (Supplementary Table 2): in Turkey near Adana, in Germany near Stuttgart, in Ukraine near Potash, in the Netherlands at Wageningen, in the United Kingdom near Aberystwyth and in Russia near Moscow. For the remainder of this paper, the sites are referred to by the name of the nearest town.

The field trials were established on arable or horticultural land except in Aberystwyth, where the trial was planted on marginal (low quality) grassland (Supplementary Table 2). At each site soil preparations suitable for the planting of cereals were made, removing the previous crop/vegetation and associated weeds. At each location the trial was planted as a randomized complete block design comprising three replicate blocks each containing a single plot of each of the 15 genotypes. Each plot measured 5 × 5 m and contained 49 plants in a 7 × 7 grid with a planting density of 1.96 plants m^−2^. The total trial area at each site was 75 × 43 m.

In 2012, soil samples were taken before planting and fertilization from two randomly selected plots in each replicate block at each location. Soil samples were collected at the 0–30, 30–60, 60–90 cm layers where there was sufficient profile depth. Samples were analyzed for pH, plant available nitrogen (N_min_) and total potassium (K), phosphorous (P), and magnesium (Mg) (Supplementary Table 3). The plant available nitrogen was determined by using CaCl_2_ extraction followed by FIA measurement (DIN ISO 14255:1998-11). Determination of soil P and K was carried out by using CAL extraction followed by flame photometer or FIA measurement (OENORM L 1087:2012-12-01). Soil pH was determined by using a glass electrode after CaCl_2_extraction (DIN ISO 10390:2005; Ehmann et al., 2017). Further inter-row soil cores were taken from each plot in October 2012 using a soil column cylinder auger (Eijelkamp, Giesbeek, Netherlands) to determine soil bulk density, soil depth, and stone content (Supplementary Table 3).

### Trial management and climatic conditions

*Miscanthus* plugs were planted by hand in May 2012 except in Adana where the trial was established earlier, in mid-April, to avoid dry and hot weather whilst planting. In spring 2012, fertilizer was applied at all the sites at rates 44 and 110 kg ha^−1^ year^−1^ P and K, respectively, which, combined with residual soil nutrients, designed to match crop requirements (Lewandowski et al., 2000). No nitrogen fertilizer was applied in the first year to minimize weed growth. From year 2 fertilizer was applied at the rate of 140 kg ha^−1^ K, 100 kg ha^−1^ P, and 60 kg ha^−1^ N applied once per season in spring, rates designed to ensure non-limiting crop nutrition at all sites.

From 2013, continuous drip irrigation was applied in Adana to compensate for lack of rainfall and to maintain the trial during prolonged drought periods. Irrigation was applied more often and in larger volumes in 2013 to ensure crop establishment and then reduced in 2014 and 2015 to identify genotypes suited to arid and hot climatic conditions. Volumes of water applied were recorded. Emerging weeds were removed regularly by hand during the growing seasons 2012–2014 at all sites.

Climate data (rainfall, air, and soil temperature and radiation) were obtained from the weather stations at the study sites. Supplementary Table 4 summarizes climatic conditions during each growing season at each location and the irrigation applied in Adana.

### Measurements

Plant survival was recorded in May 2013 as the number of plants producing new shoots in spring. Plant loss was calculated as the number of non-shooting plants expressed as a percentage of the total plants planted per plot. Any gaps occurring due to overwinter mortality in the first winter were filled in using plants from the adjacent replacement plots planted for this purpose at each corresponding site in 2012.

At the end of the third growing season (autumn 2014) canopy height was measured and stem number per plant (only stems reaching at least 60% of canopy height) was recorded on 3–5 central plants per plot.

Each year biomass was harvested from the core square (9 plants; middle 2 m^2^) of the plots in February–April depending upon location and when the crop was dry. Cutting height for yield determination was 5 cm above the soil surface. Harvested plant material was dried to constant weight at 60°C. Dry matter yield was calculated as tons of dry matter (DM) ha^−1^. Total DM yield was calculated as the sum of the plot yields over four growing seasons.

### Statistical analyses

All statistical analyses were performed with the aid of GenStat (Version 18.2; VSN International Ltd., Hemel Hempstead, UK; Payne et al., 2015). Within location, effects of species group on total 4-year biomass yield were assessed by analysis of variance according to the randomized block design. Yields of OPM-5–10 in seasons 3 and 4 were compared by analysis of variance as split plot in time. Effects of genotype and location and their interaction on biomass yield, plot mean values for canopy height and stem count in year 3 were assessed by residual maximum likelihood analysis and using a separate residual variance at each location. Where necessary, multiple pairwise comparisons within tables of means were accounted for by Bonferroni-adjustment of the comparison-wise type I error rate. Sensitivity of biomass yield, canopy height, and stem count of the genotypes to the six environments was assessed by modified joint regression analysis (Finlay and Wilkinson, 1963) as implemented in the RFINLAYWILKINSON procedure of GenStat (Payne et al., 2015). Stem counts were transformed to the square root scale prior to calculating plot means and prior to each analysis.

## Results

### Plant overwinter survival

At most field sites, there were few plant losses in the first winter after planting (Table 2). However, in Aberystwyth the plants did not establish well in the first year and in total 43% of the plants needed to be replaced. A possible reason for high plantlet mortality at this location may have been the weather conditions viz. cool air temperatures in 2012 and flooding at the time of miscanthus planting. Aberystwyth had the highest (727 mm, which is double the long term average) total rainfall and the lowest mean air temperature (11°C, which is 2° lower than the long term average) among the sites in the first growing season (Supplementary Table 4). This location also had the lowest DD_(base10)_ and PAR among the field trial sites in 2012 (see Supplementary Table 5), two important parameters known to influence miscanthus growth and yields (Clifton-Brown et al., 2000), which could result in weaker and smaller plants by winter.

**Table 2:** **Plant losses (% of plants planted) recorded in the field during the first winter (November 2012 until March 2013) for the 15 *Miscanthus* genotypes at six field locations**.

**Location**	**Genotype (OPM) and species group**														
	***Sac***				***Sac** × **Sin***						***Sin***				
	**1**	**2**	**3**	**4**	**5**	**6**	**7**	**8**	**9 Gig**	**10**	**11**	**12**	**13**	**14**	**15**
Adana	0	0	0	2	1	0	1	1	0	1	34	16	12	0	18
Stuttgart	4	1	0	3	2	2	0	1	0	0	0	3	2	3	4
Potash	3	3	1	2	0	3	2	1	13	2	1	8	1	4	14
Wageningen	1	0	1	0	2	0	0	0	0	1	0	0	0	0	1
Aberystwyth	59	82	45	55	44	28	29	27	32	35	35	31	50	57	39
Moscow	3	13	0	5	0	1	6	1	11	5	7	13	4	4	11

At the other locations, on average only 3% of all plants needed to be replaced after winter. The highest losses were observed with OPM-15 (a seed-propagated, *Sac* × *Sin* × *Sin* open-pollinated hybrid) where on average 10% of plants needed to be replaced (Aberystwyth site not included). The seedlings of this accession were initially slightly smaller at planting due to a slightly later sowing date than the other genotypes, which may have contributed to the higher mortality rate observed.

At the more northern sites with continental climate, Moscow and Potash, higher plant mortality was observed than in Wageningen or Stuttgart. At the two former locations some losses were observed for most of the genotypes but losses never exceeded 14% for any of the genotypes concerned. Interestingly, *M*. × *giganteus* showed no plant losses at the warmer field locations in Adana, Stuttgart, and Wageningen, but higher losses than the new *M. sinensis* × *M. sacchariflorus* hybrids at colder locations in Potash and Moscow, where the lowest minimum air and soil surface temperatures were recorded (Supplementary Table 6). In Adana, significant plant losses were only observed for some of the *M. sinensis* accessions (OPM-11, 12, 13, and 15).

### Biomass yield

#### Annual biomass yield

Annual biomass (t DM ha^−1^) yield varied depending on the growing season, trial location, and *Miscanthus* genotype. Overall, biomass yields increased with increasing crop maturity. Mean annual yields across all the sites and genotypes increased from 2.3 ± 0.2 t DM ha^−1^ from the first year of growth, to 7.3 ± 0.3, 9.5 ± 0.3, and 10.5 ± 0.2 t DM ha^−1^ from the second, third, and fourth years, respectively. The highest yielding location was Potash with the average annual yield of 9.6 ± 0.4 t DM ha^−1^. The lowest-yielding was Aberystwyth with 4.0 ± 0.3 t DM ha^−1^ of average annual yield. The highest average yields across locations and years were observed for *M*. × *giganteus* (9.9 ± 0.7 t DM ha^−1^) and interspecies hybrid OPM-6 (9.4 ± 0.6 t DM ha^−1^). Interspecific hybrids on average produced higher yields than *M. sinensis* and *M. sacchariflorus* genotypes (*p* < 0.001 for the comparison of *M. sinensis* and *M. sacchariflorus* groups with hybrids).

**Figure 2:**
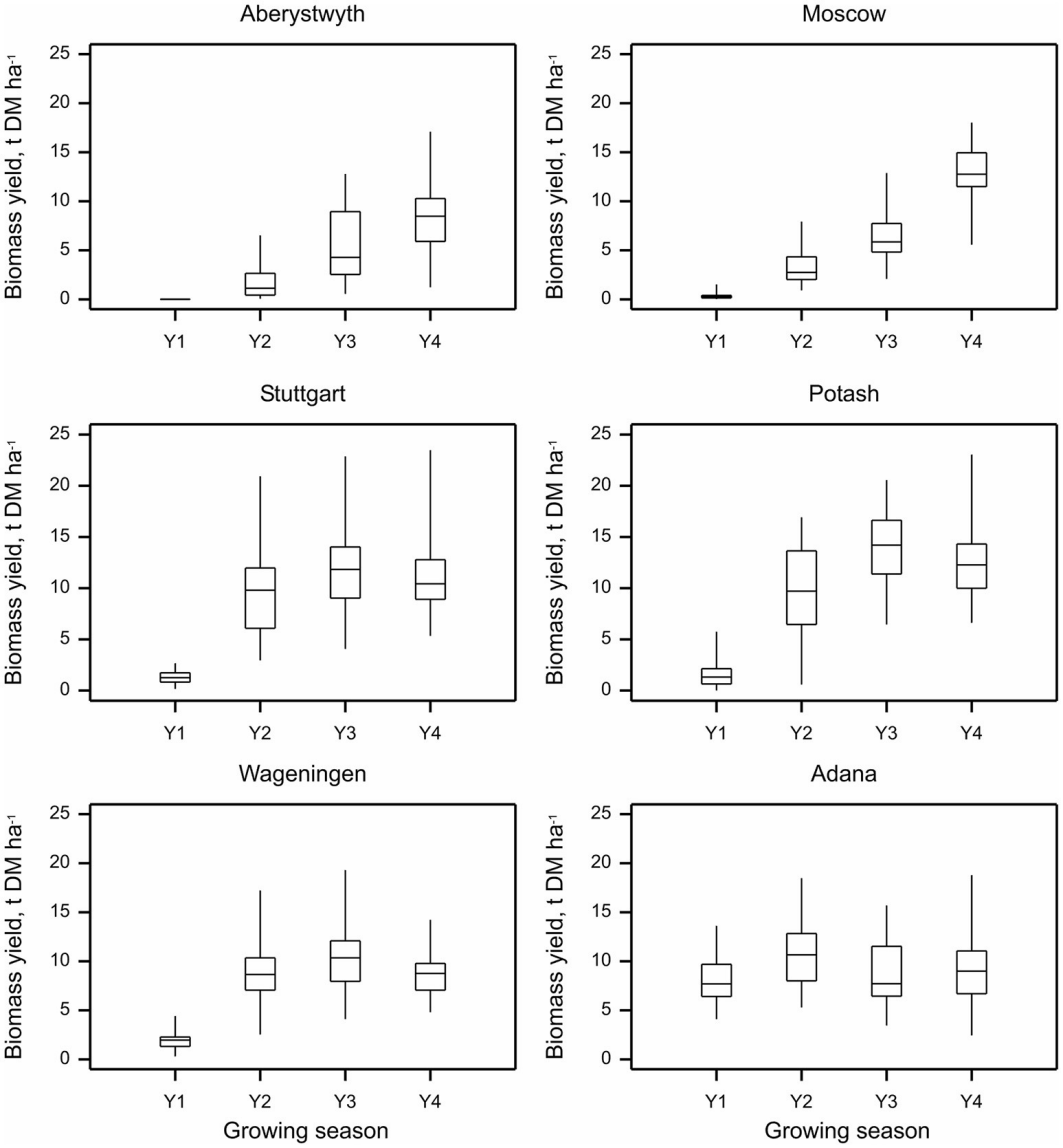
**Annual biomass yield of *Miscanthus* (15 genotypes pooled) at six trial locations over four growing seasons 2012–2015 (Y1–Y4)**. Whiskers denote the overall range at each location within each year, boxes denote interquartile ranges and within this the horizontal bar denotes the median.

At all sites except Adana annual biomass yield increased throughout the first 3 years while the crop was establishing (Figure 2). However, in Adana, high biomass yields were achieved in the first growing season. At this location, the average first-year yield reached 8.1 ± 0.4 t DM ha^−1^, 7.7 times higher than at the other sites. It increased further to 10.7 ± 0.4 t DM ha^−1^ in the second growing season and although dropping slightly in the following growing season remained relatively stable throughout seasons 3 and 4 (8.7 ± 0.5 and 9.4 ± 0.5 t DM ha^−1^ in 2014 and 2015, respectively). Interestingly, at Moscow and Aberystwyth, locations where the crop apparently took longer to establish, the yields steadily increased throughout the 4 years and possibly had not achieved their peak by year 4. At Stuttgart and Potash, good yields were achieved in the second year (9.5 ± 0.6 and 9.5 ± 0.7 t DM ha^−1^, respectively), there was however high within-site variation at these locations (Figure 2). At Stuttgart, highly variable soil depth within the site (40–100 cm) could be responsible for this variation in yield. At Wageningen and Potash, biomass yield was generally lower in year 4 than year 3 (14.1 ± 0.5 *v* 12.6 ± 0.5 at Potash, and 10.4 ± 0.4 *v* 8.7 ± 0.3 t DM ha^−1^ at Wageningen in 2014 and 2015, respectively), which was possibly due to lower rainfall in 2015 (in particular at Potash, rainfall in 2015 was almost half that in 2014; Supplementary Table 4).

In terms of biomass yield, genotypes ranked differently by year and by location. The higher-yielding genotypes were different at the six sites (see also yield ranking in Lewandowski et al., 2016). The best-yielding genotype across locations from the first growing season was *M*. × *giganteus* (OPM-9) producing on average 3.4 ± 1.0 t DM ha^−1^ and after the second and third seasons, the *sac* × *sin* hybrid OPM-6 with 10.6 ± 1 and 12.4 ± 0.9 t DM ha^−1^, respectively. In the fourth growing season, *M*. × *giganteus* showed again the highest average yield of 13.8 ± 0.7 t DM ha^−1^ across locations. Overall these two genotypes were the highest biomass producers showing either the first or the second best yield depending on the year (Table 3).

**Table 3:** **Annual biomass yield (t DM ha^−1^) of 15 *Miscanthus* genotypes at six trial locations in 2014 (Y3) analyzed by REML using separate residual variances for each location**.

**Location**	**Genotype (OPM)**															
	**1**	**2**	**3**	**4**	**5**	**6**	**7**	**8**	**9**	**10**	**11**	**12**	**13**	**14**	**15**	**Mean**
Aberystwyth	1.5	2.9	6.4	3.3	5.6	10.6	4.7	11.3	8.3	10.8	3.0	2.9	3.0	2.2	4.8	5.4
Moscow	3.4	5.5	4.7	2.9	7.2	10.4	6.8	7.6	7.8	8.5	6.2	6.0	5.6	5.7	4.3	6.2
Stuttgart	8.3	12.9	14.6	6.1	13.7	16.3	12.7	14.2	13.6	13.6	11.8	12.5	10.2	9.5	7.9	11.9
Potash	14.1	18.0	15.4	13.3	17.3	17.0	14.3	13.3	16.7	15.7	15.3	10.5	9.2	11.7	10.3	14.1
Wageningen	5.9	10.3	9.8	8.3	9.4	10.8	9.5	14.5	14.3	12.1	12.8	9.8	9.3	9.1	9.5	10.4
Adana	6.3	6.3	5.2	4.5	7.3	9.4	7.0	7.3	13.0	6.8	12.4	12.5	12.1	9.8	10.4	8.7
Mean	6.6	9.3	9.4	6.4	10.1	12.4	9.2	11.4	12.3	11.3	10.2	9.0	8.2	8.0	7.9	

*Statistical significance of effects of genotype p < 0.001 (average s.e. 0.61), location p < 0.001 (average s.e. 0.59) and interaction p < 0.001 (average s.e. 1.45)*.

At Adana, *M*. × *giganteus* was the highest-yielding genotype in the first three seasons whilst in 2015, the best yield was recorded for *M. sinensis* OPM-12. At Aberystwyth, hybrid OPM-8 consistently yielded the highest of all the genotypes in the first three seasons but in year 4 it was outperformed by *M*. × *giganteus* although not significantly so. At the other locations the best-yielding genotypes varied depending on the year (see also Lewandowski et al., 2016).

#### Total biomass yield over four growing seasons

The highest total biomass yield of 37.9 ± 1.8 t DM ha^−1^ (location mean for all genotypes) was observed at Potash, Ukraine and the second highest in Adana, Turkey (36.9 ± 1.3 t DM ha^−1^). The lowest-yielding locations were Aberystwyth with a total yield of 15.4 ± 1.3 t DM ha^−1^ and Moscow with 22.5 ± 0.9 t DM ha^−1^.

**Figure 3:**
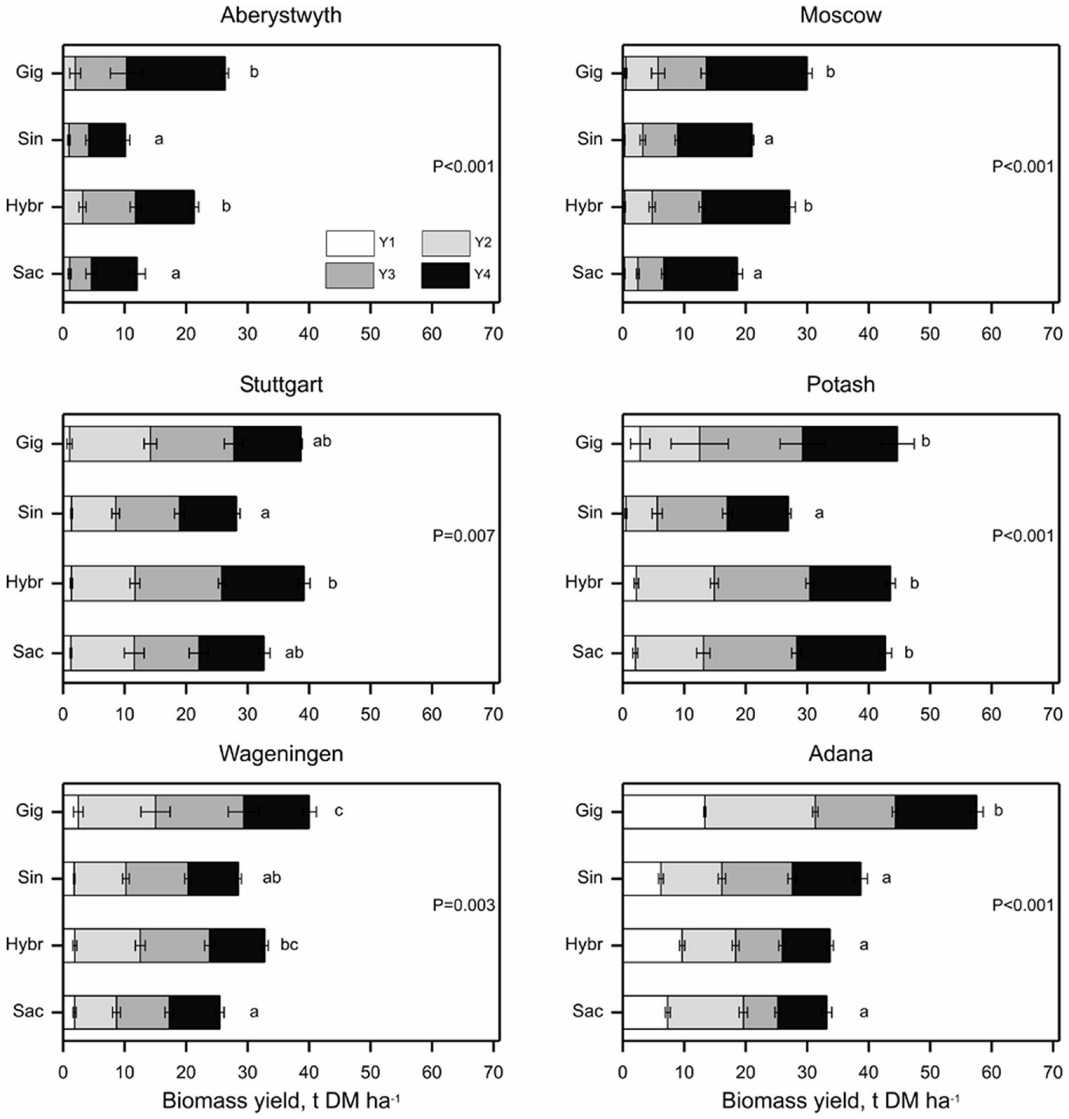
**Cumulative biomass yield over four growing seasons (Y1–Y4) at six trial locations**. *Miscanthus* genotypes were categorized as: Gig = *Miscanthus* × *giganteus*, Sin = *M. sinensis*, Hybr = *M. sinensis* × *M. sacchariflorus* hybrids or Sac = *M. sacchariflorus* genotypes. Error bars represent ± standard error of the mean for corresponding growing season. Probabilities indicate the overall effect of species group on total cumulative biomass yield within each site and differing letters indicate species group means differ (*p* < 0.05) based on bonferroni adjusted multiple comparisons.

Significant differences (*p* < 0.01) between the species groups (i.e., between “*M. sacchariflorus*,” “*M. sinensis*,” “Hybrids,” and “*M*. × *giganteus* control clone”) in total four year yield were observed at each location (Figure 3). The total yield of the new interspecies hybrids did not differ (*p* > 0.05) from that of *M*. × *giganteus* at all the locations, except Adana (the only location with additional irrigation applied), where *M*. × *giganteus* outperformed hybrids (*p* < 0.05). In particular, the hybrids OPM-6, 8, 10 achieved the same 4-year yield as *M*. × *giganteus* (locations pooled), but also one of the *M. sacchariflorus* types, OPM-2, had total yield similar to that of *M*. × *giganteus* clone. However, there was still evidence of significant differences between genotypes within species group at Aberystwyth (*p* < 0.021), Stuttgart (*p* < 0.023), and Potash (*p* < 0.01).

The *M. sinensis* types on average produced significantly less biomass than interspecies hybrids, except in Adana, where *M. sinensis* types OPM-11 and 12 produced the highest yields, and Wageningen where these two groups yielded similarly. *M. sinensis* types had on average similar total yields to *M. sacchariflorus* genotypes at all trial locations, except in Potash where *M. sacchariflorus* genotypes produced a higher total yield than *M. sinensis* types (*p* < 0.05; Figure 3). *M. sacchariflorus* on average (four genotypes pooled) produced similar to *M*. × *giganteus* yields at Potash and Stuttgart and had lower total yields than *M. giganteus* at the other locations. Over a period of 4 years, OPM-2 (*M. sacchariflorus*) and hybrid genotypes OPM-6, 8, and 10 showed similar total yields to *M*. × *giganteus* (locations pooled).

Total biomass DM yield over 4 years was linearly correlated (*p* < 0.001) with the annual yields achieved in each of the growing seasons. Over all locations the correlation increased from 0.49 in the year 1–0.90 in the second, 0.86 in the third growing seasons and 0.62 in the year 4.

#### Genotype differences in yield in an established crop (2014–2015)

**Figure 4:**
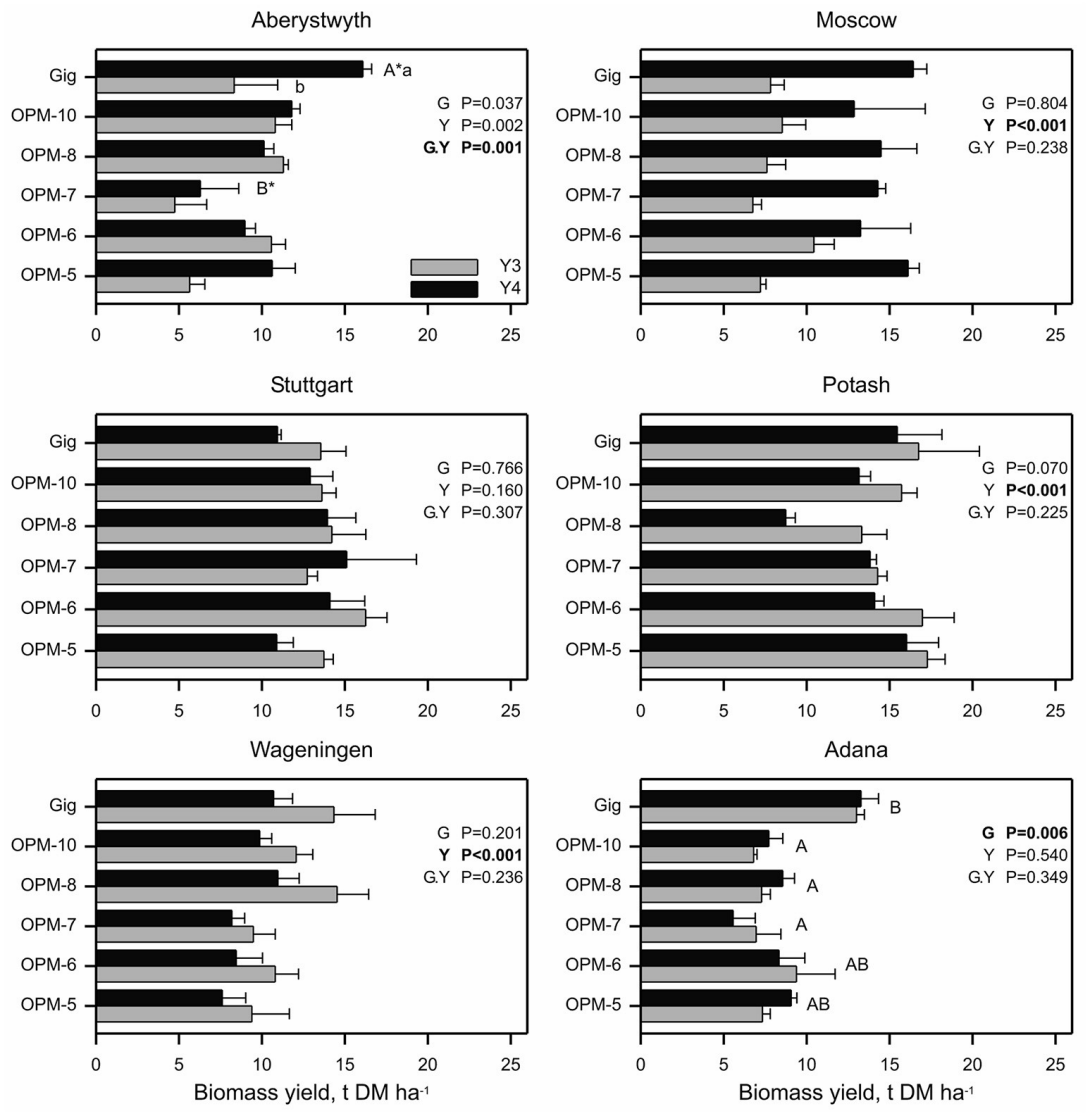
**Biomass yield of *Miscanthus* × *giganteus* and *M. sinensis* × *M. sacchariflorus* hybrids in 2014 (Y3) and 2015 (Y4) within six field trial locations**. Error bars represent the standard error of the mean. Effects of genotype, year and interaction (genotype.year) are denoted by G, Y and G.Y, respectively. At Adana, differing capital letters indicate genotype means differ (*p* < 0.05) based on bonferroni adjusted multiple comparisons. At Aberystwyth, differing capital letters (A^*^, B^*^) indicate genotype means within a year and differing lower case letters within a genotype indicate means differ between years (*p* < 0.05).

Figure 4 shows the yields of the individual interspecies hybrid genotypes and *M*. × *giganteus* in years 3 and 4, when the crop reached or approached maturity and yields stabilized. In these growing seasons there was no genotype effect on annual yield at any location except Adana, i.e., biomass yields for *M*. × *giganteus* and *Sac* × *Sin* hybrids were similar (*p* > 0.05). At Adana, *M*. × *giganteus* showed higher biomass yield than OPM-7, 8, and 10 (*p* < 0.05) while OPM-5 and 6 produced biomass yields comparable to *M*. × *giganteus*. At Potash and Wageningen year 3 biomass yields were greater than in year 4 (*p* < 0.001), which reflect differences in the weather conditions (specifically significantly decreased summer rainfall in 2015) between the years at these sites (Supplementary Table 4). At Moscow and Aberystwyth, overall mean biomass yield was affected by year (*p* < 0.001 and *p* = 0.002, respectively) and increased from year 3 to 4 indicating further crop maturation at these sites. However, at Aberystwyth the effect of year was not consistent across all genotypes with only *M*. × *giganteus* showing a significant yield increase (*p* < 0.05) between years 3 and 4. All other genotypes showed similar yield in years 3 and 4. In Stuttgart, there were no effects (*p* > 0.05) of genotype, year or of an interaction between the two.

### Canopy height and stem number

Canopy height in autumn (Table 4) was affected by site, genotype and their interaction (*p* < 0.001). On average, the tallest plants were observed in Stuttgart, Potash, and Wageningen (mean canopy height 198.5 ± 7.7, 194.4 ± 6.5, and 191.7 ± 5.0 cm, respectively) and the shortest were in Moscow (122.1 ± 3.1 cm). The genotypes of *M. sacchariflorus*, OPM-1 and 3 in particular, and *M*. × *giganteus* (OPM-9) had the highest canopy heights among all the genotypes (204.1 ± 15.6, 194.2 ± 14.8, and 212.8 ± 11.1 cm, respectively).

**Table 4:** **Season-end canopy height (cm) of 15 *Miscanthus* genotypes at six trial locations in 2014 (Y3) analyzed by REML using separate residual variances for each location**.

**Location**	**Genotype (OPM)**															
	**1**	**2**	**3**	**4**	**5**	**6**	**7**	**8**	**9**	**10**	**11**	**12**	**13**	**14**	**15**	**Mean**
Aberystwyth	168.0	112.0	173.7	141.3	146.7	161.3	139.3	186.0	180.3	142.3	111.7	151.7	103.0	107.0	114.3	142.6
Moscow	136.4	114.6	126.9	97.8	116.8	116.1	111.3	116.2	180.4	126.7	127.6	118.8	114.4	120.1	100.3	121.6
Stuttgart	253.0	228.0	246.0	190.7	162.0	173.3	207.0	173.7	234.7	243.0	175.3	220.3	170.7	152.3	147.3	198.5
Potash	286.7	250.0	261.7	191.7	181.7	165.0	176.7	175.0	221.7	185.0	198.3	161.7	161.7	163.3	136.7	194.4
Wageningen	231.7	216.7	220.0	193.3	166.7	143.3	155.0	195.0	261.7	186.7	196.7	193.3	166.7	176.7	171.7	191.7
Adana	149.0	126.0	137.0	157.3	152.0	116.3	104.7	97.3	198.0	112.7	138.0	146.3	150.0	123.3	93.3	133.4
Mean	204.1	174.5	194.2	162.0	154.3	145.9	149.0	157.2	212.8	166.1	157.9	165.4	144.4	140.5	127.3	

*Statistical significance of effects of genotype p < 0.001 (average s.e. 6.22), location p < 0.001 (average s.e. 3.95) and interaction p < 0.001 (average s.e. 13.68)*.

Stem number in growing season 3 (Table 5) was also significantly affected by site and genotype with an interaction (*p* < 0.001). Highest average stem number was observed at Wageningen (60.5 stems plant^−1^) and the lowest at Aberystwyth (27.8 stems plant^−1^). Across locations, the highest average stem number was observed for the hybrid genotypes OPM-6, 7, and 10, with 74.1, 71.2, and 68.7 stems plant^−1^, respectively. The lowest average stem numbers were observed in *M*. × *giganteus* (OPM-9; 29.1 stems plant^−1^) and OPM-2, 1, 12, and 11 (33.6, 35.1, 35.5, and 37.3 stems plant^−1^, respectively). *M. sacchariflorus* genotypes tended to have lower stem numbers than *M. sinensis* types.

**Table 5:** **Season-end stem count (stems plant^−1^) of 15 *Miscanthus* genotypes at six trial locations in 2014 (Y3) analyzed by REML using separate residual variances for each location**.

**Location**	**Genotype (OPM)**															
	**1**	**2**	**3**	**4**	**5**	**6**	**7**	**8**	**9**	**10**	**11**	**12**	**13**	**14**	**15**	**Mean**
Aberystwyth	29.2	12.6	26.5	35.5	32.1	58.6	47.8	33.8	22.0	33.8	11.2	31.4	12.7	19.7	30.2	27.8
Moscow	57.6	34.7	39.4	40.1	58.7	99.3	72.8	64.7	35.1	81.3	42.0	43.1	48.9	53.3	44.9	53.1
Stuttgart	26.1	42.6	34.6	73.8	63.3	105.9	93.8	71.5	29.8	70.1	43.2	33.5	59.9	60.5	74.6	56.6
Potash	31.4	34.9	38.0	35.7	45.3	40.7	77.6	48.3	23.9	73.2	21.3	13.5	21.4	30.8	19.9	35.1
Wageningen	23.3	32.0	38.6	54.2	39.6	116.1	93.3	66.9	25.0	91.3	68.0	44.3	102.9	67.5	98.3	60.5
Adana	49.5	52.5	54.1	42.7	39.6	43.5	49.6	36.5	41.2	70.9	54.7	55.8	43.2	36.6	32.0	46.4
Mean	35.1	33.6	38.1	46.1	45.8	74.1	71.2	52.5	29.1	68.7	37.3	35.5	43.6	43.0	46.4	

*Statistical significance of effects of genotype p < 0.001 (average s.e. 0.27; s.e. applies to means on square root scale), location p < 0.001 (average s.e. 0.31) and interaction p < 0.001 (average s.e. 0.66)*.

There was also a site × genotype interaction observed for stem number (*p* < 0.001). Based on analysis of variance within each location, genotypes differed in stem number at the field sites in Moscow, Potash, Stuttgart, and Wageningen (*p* = 0.01, *p* = 0.001, *p* < 0.001, and *p* < 0.001, respectively). At Wageningen and Moscow, OPM-6 had the highest stem numbers among the genotypes tested (Table 5). At Stuttgart, OPM-6 and 7 were the genotypes with the highest stem numbers. At Potash, stem number was highest in OPM-7. OPM-6, a high-yielding genotype, showed a higher (*p* < 0.05) number of stems compared to *M*. × *giganteus* at three locations: in Stuttgart, Wageningen, and Moscow. At two sites, Aberystwyth and Adana, no significant differences (*p* = 0.517 and *p* = 0.877, respectively) in stem number between genotypes were detected.

In the combined data set over all locations there was a positive linear correlation between biomass yield (t DM ha^−1^) and both autumn canopy height (cm) and stem number (stems plant^−1^) in the third growing season (2014). Canopy height was more strongly associated (Pearson *r* = 0.55, *p* < 0.001) with yield than stem number (*r* = 0.21, *p* < 0.001). Stem number and canopy height showed no association (*r* = 0.03, *p* = 0.649). But there were also exceptions within the genotype, in particular, OPM-6, one of the highest yielding genotypes in years 3 and 4, had a low canopy height but a high stem count.

### Phenotype sensitivity to location

Both canopy height and stem number measured in year 3 showed significant differences in sensitivities across the six locations (*p* = 0.007 and *p* = 0.01, respectively).

**Figure 5:**
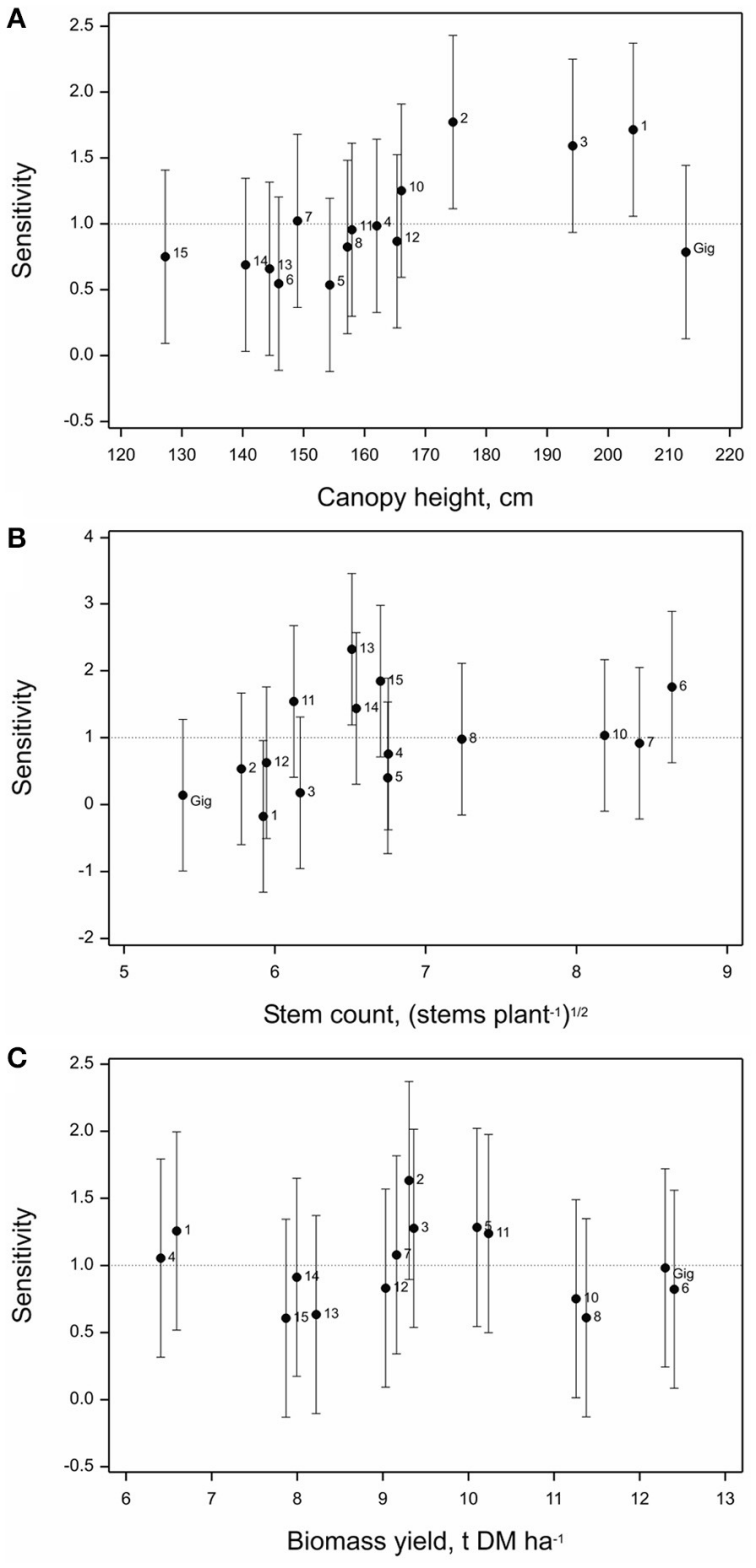
**Sensitivity of (A)** canopy height, **(B)** stem count, and **(C)** biomass yield of 15 *Miscanthus* genotypes to location in 2014 (Y3) based on joint regression analysis (Finlay and Wilkinson, 1963). Labels 1–8, Gig, 9–15 denote OPM-1 to OPM-15, respectively, vertical bars denote 95% simultaneous confidence intervals for each sensitivity estimate and the horizontal dotted line denotes the overall mean sensitivity of all 15 genotypes.

In terms of canopy height genotypes OPM-2 and 1 were most sensitive, i.e., less stable across locations than overall mean sensitivity in the data set (Figure 5A), followed closely by OPM-3 (all three belong to *M. sacchariflorus* species). The lowest sensitivities were observed for OPM-6 and 5, *Sac* × *Sin* hybrids, i.e., these genotypes had the most consistent canopy heights irrespective of the environment they were planted in.

For stem number, OPM-6, with the highest overall mean stem count, showed a higher than average sensitivity to location (tended to be less stable) than *M*. × *giganteus* and other genotypes with lower stem counts, e.g., OPM-1–4 *M. sacchariflorus* genotypes (Figure 5B). These tended to be the most stable. OPM-13 (*M. sinensis*) and OPM-15 (an open-pollinated *Sac* × *Sin* × *Sin* hybrid), showed the least stable stem counts across locations, whereas for all the *M. sacchariflorus* genotypes rather low sensitivity values have been obtained. Among the hybrids OPM-5 and among the *M. sinensis* types OPM-12 showed lower sensitivities.

Biomass yield estimated in year 3 showed no significant difference in sensitivity across the six locations (*p* = 0.269). Overall, OPM-2 tended to be the least stable and OPM-8 the most stable genotype (Figure 5C). The high-yielding *Sac* × S*in* hybrids OPM-6 and 7 showed higher than average yield sensitivity and this tended to be higher than that of *M*. × *giganteus*. Overall, all the *M. sacchariflorus* genotypes showed higher than average sensitivity, whereas most of the *M. sinensis* types tended to have lower than average sensitivity in yield to the locations studied. OPM-8, 13, and 15 had a similarly low yield sensitivity to *M*. × *giganteus*.

## Discussion

### Establishment and survival

In our experiment, the small plugs produced by *in vitro* tillering and seed were shipped to all the sites in boxes and were watered at planting. Several liters of water were applied to wet the soil in the immediate vicinity of the plug plant. This helps establish the hydraulic contact needed to prevent plug dehydration in the first 10 days while roots grow out of the plug into the soil. In most of the locations, transplanting success rates were close to 100%. The exception was Aberystwyth, where the shallow soils (Supplementary Table 2) were too damp to create a fine tilth and the soil tilth was too “lumpy” to ensure a good hydraulic contact. Further, immediately after planting in Aberystwyth, there was a 2 week period of fine weather which dried the soil surface. This was followed by an exceptionally wet (double normal rainfall) weather conditions, cold (temperatures < 16°C) and overcast in June-September (half normal radiation). This combination of conditions was highly unfavorable for *Miscanthus* establishment from delicate plugs, and resulted in high establishment plant losses. It was not our intention to make an in depth study of the agronomy of plant plug establishment as this was the task for the upscaling trials within the same OPTIMISC project (Lewandowski et al., 2016). The lessons learnt from the Aberystwyth site in the first year are nonetheless important for the subsequent agronomic trials on the establishment of *Miscanthus* from plugs in the cool wet climates and have been taken into account in the development of commercially relevant establishment protocols where safe reliable establishment of the crop is a pre-requisite to an industry based on *Miscanthus* biomass (Michal Mos and Chris Ashman, personal communication). In Aberystwyth, the lost plants were replaced with spare plants in June 2013. Weather conditions for growth in 2013 were more favorable than 2012, and no further plant losses occurred, allowing the G × E experiment to continue with measurements from the site in Aberystwyth.

It was expected that there would be differences in overwintering in the first winter following planting, particularly in the highly continental climates of Potash in Ukraine and Moscow in Russia. In Moscow, overwinter mortality was slightly higher than at most other locations (except Aberystwyth), which could be related to shorter growing season, spring frosts, and earlier low temperatures in autumn at this location. Earlier work indicated that there is a threshold (in terms of lethal temperature to kill 50% of the rhizomes, LT_50_) for overwinter freezing tolerance of the rhizomes of approximately −3.5°C for *M. sacchariflorus* and *M*. × *giganteus* (Clifton-Brown and Lewandowski, 2000). Interestingly, a repeat of an earlier freezing experiment within OPTIMISC project by partners in Belgium confirmed the −3.5°C LT_50_ (Fonteyne et al., 2016a,b). Unexpectedly, *M*. × *giganteus* survived in all sites, even in Moscow and Ukraine, where winter soil temperatures would normally have fallen below −3.5°C sometime within the 4 year trial period (between 2012 and 2015). In fact soil temperatures did not fall below −3.5°C at any of the sites, and consequently only low overwinter losses were recorded in Moscow and Potash. Some of the plant losses in Aberystwyth did occur overwinter, despite the fact that winter soil temperatures at 5 cm depth remained above freezing. The high establishment losses in Aberystwyth were more likely to be caused by the poor first season summer growing conditions which resulted in insufficient rhizome growth to overwinter, a problem seen in trials in Ireland over a decade ago (Clifton-Brown et al., 2015). In the OPTIMISC multi-location trial we did not measure the rhizome mass after the first growing season as we had done in an earlier trial (Clifton-Brown and Lewandowski, 2000) because this would have left unwanted gaps in the plots.

Adana (Turkey) provided the most exceptional environment in this experiment for early establishment. Here, without irrigation *Miscanthus* could not establish. However, with the application of irrigation amounts to almost completely cover potential evapotranspiration in the first year, the establishment rate was so rapid that many genotypes almost reached mature “ceiling” yields in a single growing season. In the Netherlands, where the soil has a light sandy texture, mature ceiling yields appear to have been reached by the end of the second year. In contrast, despite the favorable growing season temperatures and rainfall in Stuttgart, the mature yields were only attained by year 3. We believe this slower establishment is partly due to the heavy clay soil and highly variable soil depth (40–100 cm) across the site which impede rapid root and rhizome growth, In Ukraine, where the soil conditions were the best of all sites, and summer temperatures are favorable, yields increased consecutively until the third year but were reduced slightly in year 4, due to significantly decreased summer rainfall. In contrast, yields in the Aberystwyth and Moscow sites rose slowly in the first and second years, but by the third and fourth year the difference in annual productivity between sites that established most quickly (Adana and Netherlands) had begun to narrow. It will require a further year or two to ascertain if indeed the ceiling yield was reached in fourth year in Aberystwyth and Moscow.

Interestingly, as the annual productive differences between the slower and faster establishing sites reduced with stand age, the yield differences between the sites over the crops lifespan of 12–20 years (Lesur et al., 2013) would be expected to narrow. We would expect significant differences in long-term yields of the different germplasm types would be detected if yield measurements could continue.

### Yield performance and environment

The continental climate with warm summers, combined with nutrient-rich deep soils ensuring a good water supply throughout the growing season in Ukraine resulted in the highest ranked productivity of all the six sites over the first 4 years.

At Adana in Turkey, high yields could be achieved already in the first growing season and further yield increase was rather slow. A number of factors could contribute to high yields at this site. The trial in Adana was irrigated, evidently providing sufficient soil moisture content to allow successful and quick plant establishment. The Adana site had the highest PAR and degree-days (DD_base0_, _base10_) over the first growing season, and also the highest air and deep soil (over 2 m depth) temperatures among all the locations (Table 2; Supplementary Tables 2–6). The warm climate and long vegetation period seem to be advantageous for miscanthus yields at this site, when sufficient water supply was ensured. The literature sources report that *M*. × *giganteus* is providing higher yields in warmer, wetter areas with moderately heavy soils (Beale and Long, 1995; Lewandowski et al., 2000).

At two locations, in Aberystwyth and in Moscow, the yields were low in the first year after planting but continued gradually increasing over all the 4 years. The crop has possibly not yet achieved its peak yields at these two locations. As mentioned above, in Aberystwyth the weather in the first growing season directly after planting was most probably the key factor affecting the establishment and the first-year biomass yield. The total yield achieved at this location over 4 years was also the lowest among the trials. It is worth mentioning that the field trial at Aberystwyth was established on marginal, shallow soil poor on nutrients (Supplementary Tables 2, 3), on a former grassland, whereas the other trials were placed on arable or horticultural land.

The yields at Moscow site were comparable to the other sites and improved significantly in the years following establishment, reaching 16 t DM ha^−1^ for some genotypes (e.g., *M*. × *giganteus*) in year 4. Lower than expected overwinter mortality and good mature biomass yields at this site might be related to relatively mild winter soil temperatures in the years of assessment and deep snow cover preventing rhizome damage overwinter. Although air temperatures at this site (as well as in Potash in Ukraine) sometimes went lower than −20°C, soil temperature did not fall lower than 0.7°C at 20 cm depth in the first winter (Table 2). Deep soil and good plant available nitrogen supply at this site could also be advantageous for biomass production (Supplementary Tables 2, 3).

*M*. × *giganteus* gave its best yields at the sites with rich deep soil, such as Potash, or in a warm climate under sufficient irrigation, such as in Adana. *M. sinensis* genotypes on average showed their best yields in Adana, possibly profiting from a long vegetation period. Earlier, Robson et al. (2012) reported that *M. sinensis* genotypes may remain green for longer period than *M. sacchariflorus* genotypes.

Biomass yields were lower at Wageningen and Potash in the fourth growth season compared to the third. This could be a result of lower precipitation at these sites in the year 4, but also the other climate factors could play a role. Precipitation during the growing period is mentioned as the key factor for high miscanthus yields in the literature (Ercoli et al., 1999; Richter et al., 2008; Gauder et al., 2012). Some other factors, such as heat sum during the growing period, soil moisture and PAR, are also known to be important for biomass production (Gauder et al., 2012; Larsen et al., 2016). At Adana, the biomass yields dropped slightly in the last two growing seasons compared to the second which most probably was caused by the reduction in irrigation.

### Genetic variation and performance of the genotypes across sites

Across all sites over 4 years, the rankings of the most productive genotypes/hybrids were quite similar and we found less environmental specificity than expected despite the wide climatic range of the six sites. Unexpectedly, *M*. × *giganteus* survived in all sites and by the third and fourth years was amongst the highest yielding types and is a key “generic high performing genotype” with wide climatic adaptability.

The interspecies hybrid group produced more biomass than both the *M. sacchariflorus* and *M. sinensis* groups. This confirms the importance of interspecies crosses to achieve the highest yields. Overall, *M*. × *giganteus* was the highest yielding clone and OPM-6 hybrid came a close second. The low environmental specificity was a surprising result, since we expected that there would be a greater requirement for matching germplasm types to cope with environmental extremes of overwinter cold in Ukraine and Moscow and drought and heat in Adana. The relatively early senescing clone, OPM-10, was a consistent “performer” across all sites, but never the highest yielding type in any location. OPM-10's environmental resilience is noteworthy because resilience is key to production and survival in marginal land types where extremes of drought, sometimes combined with low temperatures in and out of the growing season, limit the production of food crops.

When we set up the multi-location trial in 2012, we expected the warm summers in Adana would cause similar stunting effects to those observed in Texas (Charlie Rodgers, personal communication). In fact *M*. × *giganteus* performed much better than expected. From this we conclude that *Miscanthus* × *giganteus* is still within its range of thermal adaptation in Adana and that the growing season water availability is the main constraint for production in southern Mediterranean climate, rather than heat stress. Interestingly, with reduced irrigation levels in the third and fourth growing seasons in Adana, the water saving strategies of the *M. sinensis* types detected in earlier experiments (Clifton-Brown et al., 2002), were confirmed by the significant jump in yield rank (in particular OPM-13). As irrigation water is expensive, maximizing the biomass production through improved water use efficiency is very important and a subject of intense research in several interrelated research projects, of which EU FP7's WATBIO (Taylor et al., 2016) is one of the most comprehensive including genomics for breeding.

The relatively low environment sensitivity in many selections, have both advantages and disadvantages for further breeding. A key advantage is that leading selections made in plot trials in “central” locations such as Braunschweig in Germany (with cold continental winters, warm summers with regular water deficits) have wide relevance for the selection of novel germplasm for much of Europe.

### Yield traits

Across all sites and all genotypes in 2014, there were significant positive correlations between harvested yield and autumn canopy height and stem number. For this set of germplasm, canopy height (*r* = 0.55) appeared to be more predictive for the biomass yield than stem number (*r* = 0.21). Although, these correlations were statistically significant they explained only a minor part of the observed variation in yield. In particular, OPM-6 hybrid, one of the highest yielding genotypes, had a low canopy height but a high stem count compared to the other genotypes.

A number of studies have reported correlations between yield and various morphological and physiological parameters in miscanthus (Jeżowski, 2008; Gauder et al., 2012; Robson et al., 2013; Maddison et al., 2017). Several earlier studies showed that tillering is among the most important traits influencing biomass yield (Jeżowski, 2008; Nie et al., 2016). Our results have only shown a weak association between the stem number and yield for the set of germplasm evaluated. The higher stem numbers are often associated with thinner stems (Robson et al., 2013). In the same field trial we found that germplasm types with higher stem counts have lower moisture contents at harvest (*r* = −0.43, *p* < 0.001; data not shown in this manuscript). These thinner stemmed types are easier to cut and bale at harvest than those with thicker stems (Hastings et al., 2017). They however have the disadvantage that leaf shares are higher than in the tallest genotypes (such as OPM-1 and 9), which can increase the ash content (Iqbal et al., 2017). Here, it is worth mentioning that since only stems reaching at least 60% of the canopy height were counted, this measurement may underestimate the total shoot number for the *M. sinensis* genotypes (which tend to produce multiple short stems).

To date morphological characterization has largely been carried out in “spaced plant” breeding nurseries. While spaced plant nurseries are needed to handle the large numbers of genotypes to be screened in breeding, yield may or may not correlate to in plot yield performance where the individual plants are tested in “competitive” plant stands with full canopy closure. Planting densities have a very important role to play in yield determination. In our multi-location trial we decided to standardize the planting density at two plants m^−2^ for all germplasm types based on prior experience (Clifton-Brown et al., 2001). There are many complex interactions between planting density and the germplasm morphological characteristics such as height, shoot density and growing environment. Since such trials are resource intensive these experiments should only be attempted on a very few highly promising novel hybrids.

The new data from this multi-location trial confounds our efforts to identify simple ideotypes for high yield. Both short and tall morphotypes can be effective strategies. This points us back to the importance of work on whole season photosynthetic efficiency where we know interspecies hybrids such as *M*. × *giganteus* have proved outstanding at low temperatures (Beale and Long, 1995; Davey et al., 2017). This is further complicated by environmental plasticity. For example under extremely hot climate, the morphology of *M*. × *giganteus*, which expresses a dominant phenotype associated with its tall *M. sacchariflorus* parent when grown in temperate climates (with a canopy height over 3 m), changes to a more *M. sinensis* phenotype with a multitude of short thin stems and a canopy height of about 1 m.

## Conclusions

Performance of the 15 genotypes of miscanthus has been assessed across a wide range of environments in the European countries, Russia and Turkey. A number of genotypes, in particular interspecies hybrids of *M. sinensis* and *M. sacchariflorus* showed good yield potential to be used in parallel or as a replacement to *M*. × *giganteus* standard clone. In particular, *Sac* × *Sin* hybrids were high-yielding. Two of these, OPM-6 and 7 provided similar to *M*. × *giganteus* biomass yields at most locations.

Environment-sensitive genotypes, which showed high yields but low yield stability across geographic sites, such as e.g., OPM-2 (*M. sacchariflorus*) can be recommended for use in particular locations, where they are the most productive. Whereas, the genotypes providing stable yields in different environments, such as OPM-8 or 13, can be valuable for breeding programs of miscanthus. Interestingly, *M*. × *giganteus* produced high biomass yields at multiple sites and showed a high yield stability in the Finlay Wilkinson analysis. *M. sacchariflorus* germplasm types showed high yields but the yields were more vulnerable to the environmental conditions and varied among the locations. The *M. sinensis* genotypes had overall lower yields (with some exceptions) but the yields were more stable across the locations.

This multi-location trial showed that the range of miscanthus cultivation can be extended into the Eastern areas, also for the standard clone *M*. × *giganteus* which showed good overwintering in this study. Climate changes are reducing the severity of winters, and it appears to be safe to plant *Miscanthus* further eastwards than earlier predicted, e.g., Hastings et al. (2009a,b).

## Author contributions

OK, JC, IL designed and planned the experiments; OK, CN, TV, MÖ, IT, HS performed the experiments; OK, RS analyzed the data; OK drafted the manuscript; CN, RS, IL, JC, AH, LT critically revised the manuscript draft; all the authors revised and approved the final version to be published.

## Funding

The OPTIMISC project received funding from the European Union Seventh Framework Programme (FP7/2007-2013) under grant agreement No. 289159.

### Conflict of interest statement

The authors declare that the research was conducted in the absence of any commercial or financial relationships that could be construed as a potential conflict of interest.
