# Supplementary material for: Extending Miscanthus Cultivation with Novel Germplasm at Six Contrasting Sites
Source: Front Plant Sci. 2017 Apr 19;8:563. doi: 10.3389/fpls.2017.00563 (PMC5395641; doi:10.3389/fpls.2017.00563)
Supplement: Supplementary file 1 [file Table1.pdf]

Supplementary Table 1. Information on the source species (parents for hybrids) and climate in which germplasm was collected for the 15 genotypes of miscanthus used in the multi-location trial.

**Genotype ID**	**Accession type***	**Parents if known**	**Species**	**Resul-tant species**	**Chro-mosome Nr.**	**Supplier**	**Germplasm type**	**Lat**	**Alt**	**Summer rain (mm)**	**Mean Max monthly tempera-ture**	**Mean Min monthly tempera-ture**	**Annual Degree days above 10°C**
OPM-1	wild		Sac		76	IBERS	clone	32	3	829	28.3	2.9	1734
OPM-2	wild		Sac		76	IBERS	clone	31	139	1695	27.6	8.5	1801
OPM-3	wild		Sac		76	IBERS	clone	34	22	1465	24.8	3.3	1798
OPM-4	wild		Sac		38	IBERS	clone	37	-1	550	26.3	-2.3	480
	breeders line	Mother	Sin					23	1718	1509	21.4	11.9	1803
	breeders line	Father	Sac					33	600	2020	25.1	3.2	1800
OPM-5	hybrid	Hybrid		Sin × Sac	38	IBERS	clone						
	breeders line	Mother	Sac					Unknown					
	breeders line	Father	Sin					36		1343	19.1	-6.3	826
OPM-6	hybrid			Sac × Sin	38	IBERS	clone						
	breeders line	Mother	Sac					45	112	496	20.9	-15.9	393.8
	breeders line	Father	Sin					Unknown					
OPM-7	hybrid			Sac × Sin	38	IBERS	clone						
	breeders line	Mother	Sac					Unknown					
	breeders line	Father	Sin					Unknown					
OPM-8	hybrid			Sac × Sin	38	IBERS	clone						
OPM-9	horticultural		M. × gig		57	IBERS	clone	Unknown					
OPM-10	hybrid		Sac × Sin		38	IBERS	clone	Unknown					
OPM-11	horticultural		Sin Goliath		57	IBERS	clone	Unknown					
	breeders line	Mother	Sin					37		928	23.9	-0.8	1578
	unknown	Father	Unknown					Unknown					
OPM-12	hybrid			Sin-H	38	IBERS	seed						

Genotype ID	Accession type*	Parents if known	Species	Resul-tant species	Chro-mosome Nr.	Supplier	Germplasm type	Lat	Alt	Summer rain (mm)	Mean Max monthly tempera-ture	Mean Min monthly tempera-ture	Annual Degree days above 10°C
OPM-13	BIOMIS population Cycle 2		Sin		38	WU	seed	Unknown					
OPM-14	HS-family		Sin		38	WU	seed	Unknown					
OPM-15	open-pollinated hybrid			(Sac × Sin) × Unknown	38	IBERS	seed						
	breeders line	Mother	Sac					Unknown					
	breeders line	Father	Sin					Unknown					
