# Supplementary material for: Extending Miscanthus Cultivation with Novel Germplasm at Six Contrasting Sites
Source: Front Plant Sci. 2017 Apr 19;8:563. doi: 10.3389/fpls.2017.00563 (PMC5395641; doi:10.3389/fpls.2017.00563)
Supplement: Supplementary file 2 [file Table2.pdf]

Supplementary Table 2. Latitude, longitude, altitude, soil types, previous land use and planting dates at the six locations of the field trials.

Site	Latitude	Longitude	Altitude, m	Soil type (FAO)	Previous land use	Planting date
Adana	37	35	27	Typic Xerofluvent	arable	14.04.2012
Stuttgart	48.74	8.93	463	Vertic Eutrudept	arable	22.05.2012
Potash	48.89	30.44	237	Luvic Chernozem	arable	18.05.2012
Wageningen	51.59	5.39	10	Eutric Fluvisols	horticultural	21.05.2012
Aberystwyth	52.43	−4.01	39	Dystric gleysol	grassland	18.05.2012
Moscow	55	37	140	Stagnic Cutanik Albeluvisol (Siltic, Eutric, Ruptic)	arable	21.05.2012
