# Supplementary material for: Extending Miscanthus Cultivation with Novel Germplasm at Six Contrasting Sites
Source: Front Plant Sci. 2017 Apr 19;8:563. doi: 10.3389/fpls.2017.00563 (PMC5395641; doi:10.3389/fpls.2017.00563)
Supplement: Supplementary file 3 [file Table3.pdf]

Supplementary Table 3. Soil characteristics at the locations of the field trials.

**Location**	**Soil depth, cm**	**pH**	**Total K** kg hectare^{-1}	**Total P** kg hectare^{-1}	**Total Mg** kg hectare^{-1}	**Nmin** kg hectare^{-1}	**Bulk density** kg m^{-3}	**Stone fraction**
Adana	>200	7.7	669	66	911	181	1.52	10%
Stuttgart	40-100	7.74	609	296	1,371	87	1.46	9%
Potash	>200	6.5	363	240	1,470	113	1.43	0%
Wageningen	40-100	5.88	359	403	331	45	1.43	0%
Aberystwyth	30-50	5.56	126	46	171	31	1.41	35%
Moscow	>200	4.9	148	127	772	254	1.56	3%
