# Supplementary material for: Extending Miscanthus Cultivation with Novel Germplasm at Six Contrasting Sites
Source: Front Plant Sci. 2017 Apr 19;8:563. doi: 10.3389/fpls.2017.00563 (PMC5395641; doi:10.3389/fpls.2017.00563)
Supplement: Supplementary file 4 [file Table4.pdf]

Supplementary Table 4. Mean air temperature and rainfall at the field sites in six countries for the period April to September in 2012-2015, the long term air temperature mean for this period (over 10 years data during last 30 years), and the annual means.

**Location**	**Air temperature**					**Rainfall (total)**				
	Long-term	2012	2013	2014	2015	Long-term	2012	2013	2014	2015
Adana	26.1	25.01	25.30	25.22	25.3	75.4	133	156(233*)	246(134*)	104(161*)
Stuttgart	16.4	14.90	14.36	14.75	15.4	378.8	394	622	524	315
Potash	18.5	18.50	16.92	16.96	17.6	300.2	281	329	401	215
Wageningen	15.8	14.39	14.27	15.19	14.4	376.2	448	320	501	431
Aberystwyth	13.8	11.95	12.60	13.40	12.3	401.2	727	414	231	529
Moscow	14.8	15.51	-	15.50	14.7	347.0	389	609	237	462

(*) – Amount of irrigation applied additionally in Adana, Turkey.
