# Supplementary material for: Extending Miscanthus Cultivation with Novel Germplasm at Six Contrasting Sites
Source: Front Plant Sci. 2017 Apr 19;8:563. doi: 10.3389/fpls.2017.00563 (PMC5395641; doi:10.3389/fpls.2017.00563)
Supplement: Supplementary file 5 [file Table5.pdf]

Supplementary Table 5. The degree-days (DD) and photosynthetically active radiation (PAR) at the six locations from the date of planting until 1st November 2012.

**Location**	**PAR (GJ/m^2)**	**$DD_{(base0)}$**	**$DD_{(base10)}$**
Adana	1770	5501	2790
Stuttgart	1020	3179	1118
Potash	1664	3840	1712
Wageningen	1175	2863	979
Aberystwyth	958	2888	593
Moscow	1140	3060	1200
