# Supplementary material for: Extending Miscanthus Cultivation with Novel Germplasm at Six Contrasting Sites
Source: Front Plant Sci. 2017 Apr 19;8:563. doi: 10.3389/fpls.2017.00563 (PMC5395641; doi:10.3389/fpls.2017.00563)
Supplement: Supplementary file 6 [file Table6.pdf]

Supplementary Table 6. Air and minimum soil temperatures recorded at the soil surface and at 5−20 cm depth in the field during the first winter (from November 2012 until March 2013) for the 15 miscanthus genotypes at six field locations.

	**Temperature, ^{0}C**						
**Location**	**Min air $_{2m}$**	**Average air**	**Min soil $_{surf}$**	**Min soil $_{5cm}$**	**Min soil $_{10cm}$**	**Min soil $_{20cm}$**	**Min soil $_{50cm}$**
Adana	−1.4	11.8	−4.6	6.1	7.9	NA	NA
Stuttgart	−14.1	0.3	−9.5	−4.4	NA	NA	NA
Potash	−20.3	−3.1	−19.3	NA	0.0	1.7	0.6
Wageningen	−13.0	3.1	−6.4	−0.4	NA	NA	3.4
Aberystwyth	−5.0	5.3	0.6	1.0	1.6	NA	NA
Moscow	−23.5	−4.8	−29.0	NA	NA	0.7	NA

NA − data not available for this location.
